# Extensive Longitudinal Transverse Myelitis Temporally Related to the Use of AZD1222, AstraZeneca COVID-19 Vaccine: Cerebrospinal Fluid Analysis and Recent Data Review

**DOI:** 10.1155/2022/8999853

**Published:** 2022-06-10

**Authors:** Paulo Diniz da Gama, Tiago Gomes de Alcantara, Rafaela Ramos Smaniotto, Penélope de Lima Petuco, Wammer Alves de Almeida, Rodrigo Assad Diiniz da Gama, Yara Dadalti Fragoso

**Affiliations:** ^1^Faculty of Medical Sciences of Sorocaba, PUC-SP - Campus Sorocaba, São Paulo, Brazil; ^2^The Conjunto Hospitalar de Sorocaba, Sorocaba, Brazil; ^3^Universidade Metropolitana de Santos, Santos SP, Brazil

## Abstract

While mass immunization against coronavirus disease 2019 (COVID-19) rolls out around the globe, safety concerns and adverse events that need prompt evaluation are also emerging. Neurological complications such as transverse myelitis raise concerns as cases were observed in clinical trials. Cerebrospinal fluid analysis is routine in these cases and the characteristics of the abnormalities found are of great help not only in establishing the diagnosis but also in understanding this rare condition. We present a case of extensive longitudinal transverse myelitis after vaccination with AZD1222, AstraZeneca COVID-19 vaccine, which was the first case reported in Brazil. The abnormalities found in the study of the cerebrospinal fluid in our case are reported and discussed using data from recent publications.

## 1. Introduction

Coronavirus disease 2019 (COVID-19), caused by severe acute respiratory syndrome coronavirus 2 (SARS-CoV-2) was declared to be a pandemic on March 11, 2020, by the World Health Organization [1]. Up to the present moment, several neurological symptoms and complications related to SARS-CoV-2 have been reported, irrespectively of disease severity [2–4]. In order to combat this pandemic disease, vaccines for COVID-19 have been developed at an exceptional rate. Therefore, due to the emergency situation of needing to hold back the pandemic, there are no medium to long-term studies on the efficacy and possible adverse events of these vaccines [5]. Here, we report a case of longitudinal extensive transverse myelitis (LETM) that appeared 18 days after the patient received the first dose of COVID-19 vaccine (AZD1222, AstraZeneca). We consider that the temporal relationship between the vaccine and the myelitis could mean a causal association. The cerebrospinal fluid (CSF) analyses were given with greater interest, with a subsequent review of our recent literature.

## 2. Case Report

A 47-year-old woman was admitted to our emergency unit on June 4, 2021, reporting having received the first dose of AZD1222, AstraZeneca vaccine, for COVID-19 18 days prior to her admission. Twelve hours after receiving the vaccine, she had an episode of chills, headache, retroocular pain, neck pain, tiredness, nausea, stomach pain, and shortness of breath. The symptoms diminished with symptomatic medications. After the initial improvement, the symptoms got worse again. The patient felt mild weakness in the lower limbs that evolved over 5 days until she could no longer stand up.

At the admission examination, she was conscious, with no apparent cognitive deficit, presenting motor weakness (grade 2 in the lower limbs and grade 3 in the upper limbs), hyporeflexia, bilateral Babinski's sign, mild hypoesthesia in the lower limbs without a notable sensory level, preserved coordination, and preserved function of the cranial nerves. She presented urinary retention, requiring bladder catheterization.

**Figure 1:**
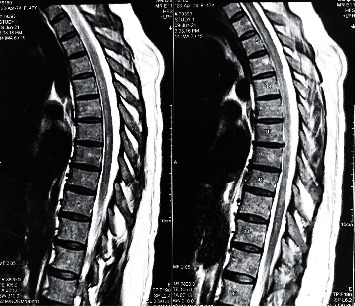
Extensive longitudinal lesion starting in C8 to the terminal cone.

**Figure 2:**
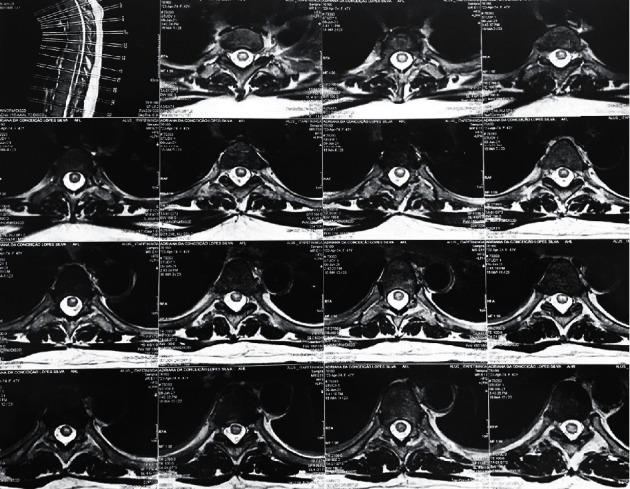
Central spinal cord lesion.

Routine blood tests were carried out at the time of her admission, and no hydroelectrolytic, metabolic, or systemic inflammatory changes were found. A magnetic resonance imaging (MRI) study on neuroaxis showed extensive spinal cord demyelination. This lesion, shown in Figures 1 and 2, was hyperintense in T2 sequence and extended from the C6 cervical level to the medullary cone. It was mildly tumefactive with no gadolinium enhancement. Brain MRI was unremarkable regarding any inflammatory process.

Cerebrospinal fluid (CSF) analysis showed a pleocytosis of 205 cells/mm³ with predominance of lymphocytes (88%), and also eosinophils (7%), neutrophils (4%), and monocytes (1%). There were increased protein levels (56.7 mg/dL) and normal levels of glucose (56 mg/dL) and chlorine (125 mEq/L). Isoelectric focusing of CSF protein and serum showed identical oligoclonal IgG bands (OCB) in both (type 4 pattern), suggestive of systemic inflammation. Testing for anti-aquaporin 4 antibody and anti-MOG antibody was negative. A nasopharyngeal swab was negative for SARS-CoV-2 in RT-PCR.

The patient received methylprednisolone 1.0 g intravenously per day for five days and symptomatic medications. She showed a gradual improvement in her motor disability and was discharged for outpatient follow-up after seven days of hospitalization. Oral methylprednisolone was prescribed at a dose of 60 mg/day, which continued in use over a 45-day gradual withdrawal regimen.

On June 17, 2021, she came back to our outpatient clinic for reassessment of her condition and reported having had gradual improvement in her motor deficit. She was already walking with little disability. She was evaluated again on July 29, 2021, showing some further improvement with strength recovery in all limbs, but still showing exacerbated deep tendon reflexes with bilateral positive Babinski's sign.

## 3. Discussion

During the current COVID-19 pandemic, a growing number of neurological manifestations and complications have been described [2–4]. Most cases consisted of postinfectious immune-mediated myelitis, and only a few have been considered parainfectious or infectious [6,7]. Particularly regarding COVID-19 vaccines, a case of transverse myelitis was reported during a preapproval clinical trial for AZD1222, AstraZeneca vaccine [8]. National health surveillance boards have already received several spontaneous reports on myelitis following COVID-19 vaccines, but no further data on diagnostic findings are available and, generally, no distinction has been made between infectious and other etiologies [9]. Hence, the implication of these spontaneous reports remains vague and assessment of potential causality is not possible.

Pagenkopf and Sudmeyer [10] recently described a very well-documented case of a 45-year-old male with LETM after application of the AZD1222, AstraZeneca COVID-19 vaccine. This showed many similarities to our clinical case regarding the time of symptom onset, duration, degree of involvement of disabilities in the acute phase, and residual state of symptoms. We also noticed similarities regarding the MRI of the spinal cord in their study, which also showed a lesion with no gadolinium enhancement. In our case, the lesion was more extensive with a minor swelling effect. Their CSF evaluation also showed similarities regarding the increased protein levels and pleocytosis. However, possibly due to the different CSF collection periods, our patient did not present glucose consumption and had a predominance of lymphocytes in CSF, just like in the follow-up CSF of that reported case. In addition, our patient presented OCB in CSF and in serum (pattern 4), which were not visualized in the abovementioned case [10]. Notghi et al. [11] also described a case of a 58-year-old man who developed initial LEMT symptoms 7 days after receiving the AstraZeneca vaccine. The CSF analysis showed a large increase in proteins, lymphocyte pleocytosis predominantly in addition to the presence of OCB in the CSF and serum, pattern 4. In this description, the clinical history, with good evolution of their disabilities, the MRI and especially all data from the CSF study were similar to our case report. Other authors such as Yong Tan et al. [12] and Hsiao et al. [13]. also described cases of LEMT after receiving the AstraZeneca vaccine. The CSF study in these two cases also showed an inflammatory activity with the presence of increased proteins, but the first did not find the presence of OCB and the second did not provide this data. In these two cases, the images and the favorable clinical evolution were similar to our case. McLean and Trefts [14] reported a 69-year-old previously healthy female presented with LEMT, 2 days after vaccination with the Pfizer-BioNTech mRNA vaccine. This patient also revealed OCB in the CSF with matching bands in the serum (pattern 4) just like our case report, although other data from the CSF analysis were normal. MRI and the favorable clinical evolution also showed similarities. Ali Alshararni [15] also reported a case of a 38-year-old man who started his symptoms two days after also using Pfizer's vaccine. An increase in CSF proteins showing inflammatory activity was described, but there were no data provided regarding the number of cells and the study of OCB. Gao et al. [16] report a case of LETM that occurred two days after vaccination with the Moderna COVID-19 (mRNA-1273) vaccine, as well as Khan et al. [17] that report one day after the same vaccine. Both authors showed an inflammatory pattern in the CSF due to the increase in proteins, and the first found the presence of OCB in CSF and serum (pattern 4) without cell increase and the second described lymphocytic cell increase and did not find the presence of OCB (pattern 1). Tahir et al. [18] reported a case of a 44-year-old woman who presented LEMT 10 days after receiving the Johnson & Johnson COVID-19 vaccine. This patient presented clinical and image manifestations with similar characteristics to our reported case, including the CSF evaluation, which showed increased protein, normal glucose, and pleocytosis with an expressive lymphocytic predominance. In this case, there was an absence of OCB in CSF and serum (pattern 1). Erdem et al. [19] reported a case of a 78-year-old woman who also presented LEMT with symptom onset 3 weeks prior to receiving the second dose of CoronaVac vaccine (Sinovac Life Sciences, China). In this case, the only data that evidenced an inflammatory activity in the CSF was an increase in protein concentration.  Table 1 shows a schematic summary of all these reported cases.

Because the specific pathogenesis of postvaccinal LEMT are unknown, there is no specific test, be it lab-based or otherwise available, which is why one needs to rely on “circumstantial evidence.” The diagnosis is based on typical, yet not limited to, clinical findings, magnetic resonance imaging, and CSF analysis as well as other investigations.

Detection of intrathecal IgG synthesis is part of the routine CSF work-up [20]. Isoelectric focusing and subsequent immunoblotting is the gold standard to visualize clonally restricted IgG OCB in CSF [21]. Five different patterns of isoelectric focusing have been defined: pattern 1: normal; pattern 2: OCB IgG restricted to CSF; pattern 3: OCB IgG in CSF with additional identical bands in CSF and serum (combination of patterns 2 and 4); pattern 4: identical OCB in CSF and serum (a mirror effect); pattern 5: monoclonal IgG bands in CSF and serum (myeloma or monoclonal gammopathy of uncertain significance). The appearance of OCB in CSF without corresponding bands in serum constitute a local intrathecal synthesis of IgG and supports the diagnosis of a variety of inflammatory central nervous system (CNS) diseases extending from an autoimmune to infectious pathology [20, 21]. Apart from multiple sclerosis, there is a long list of diagnoses with OCB restricted to CSF reported: neuromyelitis optica spectrum disorder, MOG-IgG antibody associated syndromes, systemic lupus erythematosus, Behcet's disease, CNS vasculitis, acute and chronic infections, anti-NMDA and other autoimmune encephalitis, cerebral tumors including lymphomas, among others [20, 21]. Of most interest for neurological diagnosis are patterns 1, 2, and 3, because they reject (pattern 1) or confirm (patterns 2 and 3) the intrathecal IgG production. Furthermore, blood-derived OCB in CSF (patterns 3 and 4) can give additional information because it provides evidence of both systemic and intrathecal (pattern 3) or only systemic immune activation (pattern 4). Lastly, the “mirror-pattern” (pattern 4) is most frequently associated with various peripheral inflammatory neuropathies (e.g., Guillain-Barré) and very less frequently seen with systemic infections, systemic autoimmune diseases, or neoplastic disorders. Often this disease involving CNS synthesizes IgG intrathecally more common (pattern 3) [20, 21].

Of the 11 cases of LEMT post COVID-19 vaccine that we describe in this article, 4 of them had OCB with a distribution pattern 4 and no case was described OCB with other patterns of distribution, and this attracted a lot of attention.

The observation of the clinical characteristics of postvaccination LEMT, with its favorable evolution, together with the peculiar data of MRI and the analysis of the CSF, which demonstrates not only an inflammatory activity in the CNS by the presence of increased proteins and pleocytosis but also by the observation of cases with this OCB “mirror-pattern” (pattern 4) which may not only be decisive for a better understanding of the pathophysiology of postvaccination LEMT but also may now better suggest the diagnosis of this rare condition.

There was no outstanding feature regarding clinical-evolutionary aspects, MRI features, or CSF presentation regarding the types of vaccine, which could lead to suggest a different inflammatory response. What we can conclude is still very initial, due to the low number of cases reported in the literature.

Nevertheless, despite the adverse effects reported by COVID-19 vaccines, which to date can be considered rare, it is essential to emphasize that the protective effects of vaccination can still greatly contribute to the health of individuals and the world.

## 4. Conclusion

LEMT has apparently been shown to be a temporal-related effect of COVID-19 vaccines and should be considered promptness to the onset of symptoms. The study of CSF with the presence of inflammatory activity and the demonstration of the presence of OCB equally distributed in CSF and in serum may help to consider this rare condition, until new evidence is confirmed.

## Figures and Tables

**Table 1:** Characteristics of cerebrospinal fluid analysis in reported cases of longitudinal extensive transverse myelitis (LEMT) post COVID-19 vaccine.

Author/country	Type of vaccine	CSF protein mg/dL	CSF glucose mg/dL	CSF white blood cells *x* u/L	CSF OCB	Serum OCB
**Our case** Brazil	AstraZeneca	56.4	56 (123 serum)	205 (88% lymphocytes)	Positive	Positive
Pagenkopf and Sudmeyer [10] ermany	AstraZeneca	1400 7 days after normal	Decreased 7 days after normal	481 (67% neutrophil) 7 days after 76 (100% lymphocyte)	Negative	Negative
Notghi et al. [11] United Kingdom	AstraZeneca	168.0	76 (140 serum)	11 (100% lymphocytes)	Positive	Positive
Yong Tan et al. [12] Malaysia	AstraZeneca	54.6	31 (56 serum)	<5	Negative	Negative
Hsiao et al. [13] Taiwan	AstraZeneca	44.3	No data	11 (100% lymphocytes)	No data	No data
McLean and Trefts [14] USA	Pfizer	<40	Normal	<5	Positive	Positive
Ali Alshararni [15] Saudi Arabia	Pfizer	62.1	46 (- serum)	No data	No data	No data
Gao et al. [16] Taiwan	Moderna	57.2	71 (117 serum)	15 (73% neutrophil)	Negative	Negative
Khan et al. [17] USA	Moderna	56.0	77 (125 serum)	2	Positive	Positive
Tahir et al. [18] USA	Johnson and Johnson	43	71	227 (96% lymphocytes)	Negative	Negative
Erdem et al. [19] Turkey	CoronaVac	56.0	104 (150 serum)	2	Negative	Negative

## Data Availability

The data that support the findings of this paper are available from the corresponding author upon reasonable request.

## References

[1] Zhu N., Zhang D., Wang W. (2020). A novel coronavirus from patients with pneumonia in China, 2019. *New England Journal of Medicine*.

[2] Meppiel E., Peiffer-Smadja N., Maury A. (2021). Neurologic manifestations associated with COVID-19: a multicentre registry. *Clinical microbiology and infection: The Official Publication of the European Society of Clinical Microbiology and Infectious Diseases*.

[3] Ferreira Y. B., Freire F. M. R. F., Freire F. M. R. (2021). Neurological manifestations associated with COVID- 19: a review. *Brazilian Journal of Case Reports*.

[4] Chou S. H.-Y., Beghi E., Helbok R. (2021). Global incidence of neurological manifestations among patients hospitalized with COVID-19-A report for the GCS-NeuroCOVID consortium and the ENERGY consortium. *JAMA Network Open*.

[5] Health Organization W. (2021). Coronavirus disease (COVID-19): Vaccines safety. https://www.who.int/emergencies/diseases/novel-coronavirus-2019/question-and-answers-hub/q-a-detail/coronavirus-disease-(covid-19)-vaccines-safety.

[6] Artemiadis A., Liampas A., Hadjigeorgiou L., Zis P. (2021). Myelopathy associated with SARS-COV-2 infection. A systematic review. *A systematic review Neurological Research*.

[7] Román G. C., Gracia F., Torres A., Palacios A., Gracia K., Harris D. (2021). Acute transverse myelitis (ATM):Clinical review of 43 patients with COVID-19-associated ATM and 3 post-vaccination ATM serious adverse events with the ChAdOx1 nCoV-19 vaccine (AZD1222). *Frontiers in Immunology*.

[8] Voysey M., Clemens S. A. C., Costa C. (2021). Safety and efficacy of the ChAdOx1 nCoV-19 vaccine (AZD1222) against SARS-CoV-2: an interim analysis of four randomised controlled trials in Brazil, South Africa, and the UK. *Lancet (London, England)*.

[9] (2021). COVID-19 Vaccine AstraZeneca Analysis Print. https://assets.publishing.service.gov.uk/government/uploads/system/uploads/attachment_data/file/1025766/COVID-19_AstraZeneca_Vaccine_Analysis_Print_DLP_06.10.2021.pdf.

[10] Pagenkopf C., Südmeyer M. (2021). A case of longitudinally extensive transverse myelitis following vaccination against Covid-19. *Journal of Neuroimmunology*.

[11] Notghi A. A., Atley J., Silva M. (2021). Lessons of the month 1: longitudinal extensive transverse myelitis following AstraZeneca COVID-19 vaccination. *Clinical Medicine*.

[12] Yong Tan W., Yusof Khan A. H. K., Yaakob M. N. M. (2021). Longitudinal extensive transverse myelitis following ChAdOx1 nCOV-19 vaccine: a case report. *BMC Neurology*.

[13] Hsiao Y.-T., Tsai M.-J., Chen Y.-H., Hsu C.-F. (2021). Acute transverse myelitis after COVID-19 vaccination. *Medicina*.

[14] McLean P., Trefts L. (2021). Transverse myelitis 48 hours after the administration of an mRNA COVID 19 vaccine. *Neuroimmunology Reports*.

[15] Ali Alshararni A. (2021). Acute transverse myelitis associated with COVID-19 vaccine: a case report. *International Journal of Research in Pharmacy and Science*.

[16] Gao J.-J., Tseng H.-P., Lin C.-L. (2021). Acute transverse myelitis following COVID-19 vaccination. *Vaccines*.

[17] Khan E., Shrestha A. K., Colantonio M. A. (2021). Acute transverse myelitis following SARS-CoV-2 vaccination: a case report and review of literature. *Journal of Neurology*.

[18] Tahir N., Koorapati G., Prasad S. (2021). SARS-CoV-2 vaccination-induced transverse myelitis. *Cureus*.

[19] Erdem N. Ş., Seden D., Tuğba Ö. (2021). Acute transverse myelitis after inactivated COVID-19 vaccine. *Ideggyogyaszati Szemle*.

[20] Deisenhammer F., Bartos A., Egg R. (2006). Guidelines on routine cerebrospinal fluid analysis. Report from an EFNS task force. *European Journal of Neurology*.

[21] Freedman M. S., Thompson E. J., Deisenhammer F. (2005). Recommended standard of cerebrospinal fluid analysis in the diagnosis of multiple sclerosis. *Archives of Neurology*.

